# Genetic algorithm learning as a robust approach to RNA editing site prediction

**DOI:** 10.1186/1471-2105-7-145

**Published:** 2006-03-16

**Authors:** James Thompson, Shuba Gopal

**Affiliations:** 1 Department of Biological Sciences, Rochester Institute of Technology, Rochester, NY 14623, USA

## Abstract

**Background:**

RNA editing is one of several post-transcriptional modifications that may contribute to organismal complexity in the face of limited gene complement in a genome. One form, known as *C *→ *U *editing, appears to exist in a wide range of organisms, but most instances of this form of RNA editing have been discovered serendipitously. With the large amount of genomic and transcriptomic data now available, a computational analysis could provide a more rapid means of identifying novel sites of *C *→ *U *RNA editing. Previous efforts have had some success but also some limitations. We present a computational method for identifying *C *→ *U *RNA editing sites in genomic sequences that is both robust and generalizable. We evaluate its potential use on the best data set available for these purposes: *C *→ *U *editing sites in plant mitochondrial genomes.

**Results:**

Our method is derived from a machine learning approach known as a genetic algorithm. REGAL (RNA Editing site prediction by Genetic Algorithm Learning) is 87% accurate when tested on three mitochondrial genomes, with an overall sensitivity of 82% and an overall specificity of 91%. REGAL's performance significantly improves on other *ab initio *approaches to predicting RNA editing sites in this data set. REGAL has a comparable sensitivity and higher specificity than approaches which rely on sequence homology, and it has the advantage that strong sequence conservation is not required for reliable prediction of edit sites.

**Conclusion:**

Our results suggest that *ab initio *methods can generate robust classifiers of putative edit sites, and we highlight the value of combinatorial approaches as embodied by genetic algorithms. We present REGAL as one approach with the potential to be generalized to other organisms exhibiting *C *→ *U *RNA editing.

## Background

The recent completion of genomes from organisms of vastly differing complexity has highlighted a key aspect: gene number does not directly correlate with organismal complexity. While *Drosophila melanogaster *was predicted to have just over 13,000 genes [1], the current estimate for the human genome at 20,000 to 25,000 genes is barely double that [2]. To account for this apparent discrepancy, it has been postulated that post-transcriptional modifications may play a large role in the generation of complexity from the limited complement of genes available in a given genome [3].

Of the many post-transcriptional modifications known, RNA editing is perhaps the least well understood. RNA editing encompasses a variety of processes that involve the modification, insertion or deletion of nucleotides in a mRNA transcript. This can significantly alter the final protein product. Proteins may be truncated by the introduction of premature stop codons, or the protein sequence and subsequently its structure are altered. In some organisms, up to 50% of the bases in a given transcript may be edited, resulting in minimal correspondence with the original genomic template [4]. Little is known about the actual mechanisms that direct RNA editing, but instances of RNA editing appear across the eukaryotic spectrum [5,6].

Here we focus on a specific form of RNA editing in which cytosine nucleotides are deaminated to form uridines (*C *→ *U *editing). Four instances of such editing are known in humans [5], but the best data set for studying this phenomenon computationally is derived from plant mitochondrial transcriptomes [7,8]. The exact mechanism by which a given cytosine (C) is selected for RNA editing in plant mitochondrial genomes is unknown at this time. However, multiple previous reports have established the need for certain *cis *factors upstream and downstream of the edited C [7,9]. In particular, the nucleotide immediately 5' of the edited C appears to be significant and is a pyrimidine in 93% of known instances [7,10]. Other features of edited Cs include several aspects of the downstream protein product. In the original publication reporting editing sites in *Arabidopsis thaliana*, for example, Giege and Brennicke noted that the majority of edited Cs were in the second codon position. In addition, they observed that the majority of edited Cs lead to codons that encoded more hydrophobic amino acids than the pre-edited codon [10].

We used these and other features to develop a method for identifying *C *→ *U *editing sites in plant mitochondrial genomes using a machine learning approach known as a genetic algorithm (GA). We have trained our method on a subset of the known editing sites from *A. thaliana*, and tested the method on the mitochondrial genomes of *A. thaliana, Brassica napus *and *Oryza sativa*. REGAL (RNA Editing site prediction by Genetic Algorithm Learning) has a mean accuracy of 87% across three genomes (range from 86% to 88%), with a mean specificity of 91% (91% to 92%) and a mean sensitivity of 82% (81% to 85%). REGAL significantly outperforms the other *ab initio *computational methods for the identification of *C *→ *U *RNA editing sites in plant mitochondria [7]. In addition, REGAL has a comparable sensitivity, and higher specificity and accuracy than an approach that utilizes sequence homology to predict these sites [8]. We present the REGAL approach and propose some applications of the underlying method to other problems in this realm.

### Problem statement

Since the discovery of RNA editing in a variety of organisms, the key challenge has been finding instances of RNA editing in a given genome. Most instances of RNA editing have been uncovered by serendipity followed by painstaking experimental analysis [5,11]. Given the large volume of genomic and other sequence data now available for a variety of organisms, a computational approach could provide a more rapid means for the identification of new *C *→ *U *RNA editing sites. Ideally, we would seek a computational method with the ability to predict novel instances of RNA editing given a genomic sequence and some knowledge of the features of edit sites specific for that organism. An optimal method would be one that can be easily extended to the wide spectrum of eukaryotes that exhibit *C *→ *U *RNA editing. We wished to develop such a method using the best data set available, and the plant mitochondrial genomes provide a platform for demonstrating the feasibility of our approach.

There are essentially two approaches to any predictive algorithm. The first is to draw on sequence homology to identify putative sites of editing. Several computational approaches developed recently have utilized this approach. For example, insertion C editing in *Physarum polycephalum *has been modeled by reverse translating closely related protein sequences and comparing to the relevant nucleotide sequence. Any differences between the nucleotide sequence and the reverse translated protein sequence are then candidate regions for C insertion editing [12]. A similar approach, known as PREP-Mt, has been applied to the specific realm of *C *→ *U *editing in the plant mitochondrial genomes [8].

The advantage of these approaches is that it is relatively easy to identify the sites where editing must occur in order for a transcript to yield the known protein product. While such approaches can have high accuracy, they are contingent upon the availability of sequences that are highly conserved. Furthermore, such approaches can only reliably identify RNA editing sites in which an amino acid change is effected. However, a certain proportion of all RNA editing appears to alter codons without necessarily altering the downstream translation product [8,10]. These silent edit sites cannot be reliably predicted by reverse translation of protein sequences.

An alternative to reverse translation of protein sequences is to use expressed sequence tags (ESTs) and mRNAs to identify putative edit sites. Two recent approaches to identifying adenosine to inosine (*A *→ *I*) substitution editing utilized these data to identify sites of a single nucleotide mismatch. These sites were then analyzed for evidence of RNA editing using criteria such as secondary structure constraints and conservation across genomes [13,14]. Again, the advantage of such an approach is that accuracy is likely to be relatively high. However, the limitation is that a large set of transcriptomic data must be available to reliably survey all possible edit sites in a genome.

In contrast, an *ab initio *approach attempts to predict edit sites based on the intrinsic properties of the sequences being analyzed. The primary advantage is that the need for highly conserved sequences does not constrain the set of problems that can be explored. *Ab initio *methods can potentially identify edit sites even in newly identified genes and genomes, and it can identify both silent and amino acid altering edit sites with equal precision. The chief concern with *ab initio *methods is the risk of false positives, or the prediction of edit sites where none exist. However, with sufficient training data, this concern can usually be overcome.

The challenge of predicting edit sites *ab initio *is primarily one of classification. For the specific case of *C *→ *U *editing, the question is: Given a candidate genome, which cytosines are most likely to be edited? Classification problems have essentially two kinds of solutions. Linear approaches evaluate variables sequentially for their ability to accurately classify a given cytosine. A linear approach known as the classification tree was used in a previous effort to identify *C *→ *U *editing in plant mitochondrial sequences [7]. Another common linear approach, the Hidden Markov Model (HMM), is often used in such cases to generate probabilistic predictions based on given features. Such approaches have the advantage that each variable can be tested individually for its relevance to the classification problem. However, it is difficult to then combine these variables, or to assess the relative importance of one variable with respect to a similarly effective variable.

In contrast, non-linear approaches utilize combinatorial analysis to arrive at a solution to the problem at hand. We suspected that predicting *C *→ *U *edit sites would be a combinatorial problem, so we chose a non-linear approach with good results. Genetic algorithms (GAs) represent a class of function optimization techniques that are derived from observations on the genetics of natural selection. In a GA simulation, solutions to the problem at hand are represented as virtual 'organisms' whose 'genome' encodes a specific solution. Performance of virtual organisms is ranked according to a fitness function, and data on the fitness of organisms in the population is used to select organisms for mutation, breeding and death. By applying genetic operators to organisms in a manner corresponding to their fitness, the GA will converge to an optimal or near optimal solution relatively quickly [15]. Parallelism is implicit to the algorithm, and an important characteristic is the ability of genetic algorithms to search through very large sets of possible solutions [16].

### Definitions

Since the language of GAs invokes common biological terms such as genomes and chromosomes, confusion is often inevitable when GAs are applied to biological problems. Our convention here is to indicate the GA entities by the use of the following font: organism.

The initial phase of a GA implementation involves creating a population of organisms, each with an unique genome. The genome or chromosome is a string of digits that encode the variables of interest for the problem at hand. In this instance, we used a binary encoding, so the alphabet of available characters is the set {0,1}. Each organism is evaluated for its fitness. The key to a successful GA simulation is the fitness function. In this case our fitness function is the ability of a given organism to accurately classify candidate edit sites as likely edited or unedited.

A GA simulation essentially mimics evolutionary processes by creating organisms that are evaluated for fitness using the fitness function. The evaluation occurs across hundreds of iterations, or generations. In each generation, the fittest organisms are retained and allowed to crossover. In essence, the genomes of these organisms are allowed to mix and match, much as they do in a real population. Organisms that fail to meet the fitness threshold are killed off in each generation, ensuring that successful organisms dominate the "gene pool" in successive generations.

In addition, in each generation, organisms are selected for mutation. That is, a random change is introduced into the chromosome of a given organism. This step ensures that new organisms can be generated, avoiding stagnation caused by repeated recombination of a limited set of genomes. Mutation generally occurs in a probabilistic manner, but the actual changes introduced are random events. That is, some organisms may be targeted for higher levels of mutation based on their overall fitness. The actual modifications to the genomes, however, are driven by a random generator. After many iterations of mutation, crossover and fitness evaluations, one or a few organisms remain, each capable of generating highly accurate solutions to the problem at hand. Each of these organisms can now be deconstructed to better understand which variables and in what combination were required to yield the solutions of interest.

We used a genetic algorithm to identify the best solution, which we then encapsulated into the method we call REGAL. When we refer to the training phase of our analysis, we use the generic term GA because the genetic algorithm was applied to identify the best solution. We use the term REGAL to refer to the best solution identified and its use in predicting edit sites in the three mitochondrial genomes.

## Implementation

### Data

The *C *→ *U *editing process has been observed in the mitochondria of many plant species [11], but we focused on the genomic data from three: *Arabidopsis thaliana, Brassica napus *and *Oryza sativa *[10,17,18]. Sequence data were obtained from GenBank: [GenBank:NC_001284, GenBank:AP006644, GenBank:AB076665 and GenBank:AB076666] for each of the three genomes respectively.

To map edit sites to coding regions, an *ad hoc *Perl script utilizing modules from the BioPerl project [19] was used. After extracting annotated coding sequences, each edit site was assigned to a coding sequence based on the genomic coordinates for that edit site. While there is sometimes overlap between coding sequences in the plant genomes, this overlap never contained an edit site so that assignment of an editing site to a single coding sequence was unambiguous. There were several inconsistent annotations, but these were either corrected by hand to the best of our knowledge or excluded from the final data sets. The total set of all editing sites and their positions relative to the coding sequences makes up the set of true positives. We utilized 436 edit sites from *A. thaliana*, 416 from *B. napus*, and 481 from *O. sativa*. Next, a set of true negatives was defined. Unedited cytosines were randomly selected from the set of coding sequences so that the number of unedited and edited cytosines were equal (e.g, for the *A. thaliana *data set there were 436 unedited cytosines selected to go along with 436 edited sites). The full set of true positives and true negatives utilized for each genome are included (see Additional File 1).

#### Training and testing REGAL

During training, a subset of the edited and unedited sites from *A. thaliana *were utilized. Of the 872 sites (436 in each category), an equal number of edited and unedited sites were selected at random to create training and testing subsets. In each iteration of data preparation, 100 known edited sites and 100 known unedited sites were randomly selected for use as a testing subset. The remaining data were used to train the GA. The best organism from each simulation was stored in a MySQL database.

Once we had identified the most accurate organism from the set of possible organisms in the GA simulations, we applied this solution to the data from the *A. thaliana *testing data sets as well as the *B. napus *and *O. sativa *genomes. The genomes of *B. napus *and 4*O. sativa *were used solely for testing and independent validation. For each set of known edit sites in these genomes, we randomly selected an equivalent number of non-edited sites so that we could estimate measures of performance including accuracy, sensitivity and specificity.

### Designing the genetic algorithm

#### Encoding variables for the GA

**Figure 1:**
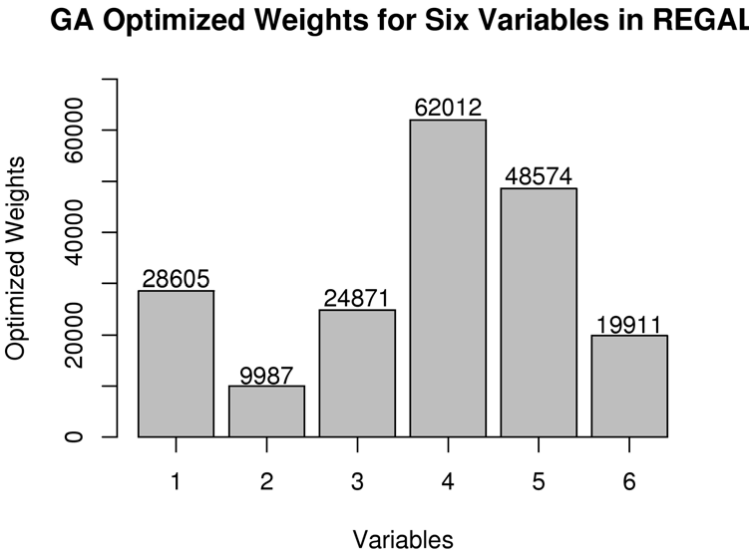
**GA optimized weights for six variables in REGAL**. We selected six variables and utilized the GA to optimize the weights for these variables. The greater the importance of a variable, the higher the value as shown here. Variables were abbreviated as follows: 1 = codon_transition, transition probability for codon pre- and post-edit; 2 = +1_nucleotide, nucleotide in +1 position relative to candidate C; 3 = hydrophobicity, likelihood that edit will yield a more hydrophobic amino acid than the unedited codon; 4 = amino_acid_transition, transition probability for amino acid pre- and post-edit; 5 = codon_position, position of the candidate edit site with respect to the codon (i.e. first, second or third position); and 6 = -1_nucleotide, nucleotide in the -1 position relative to candidate C.

For our GA, we decided to incorporate six variables based on features that had been reported to be of importance in selecting candidate cytosines for editing. These are listed in Figure 1. As discussed, several of these features were noted in previous work on RNA editing in this system [10]. We noted the codon position of the candidate C (codon_position), included whether the edited codon was more hydrophobic than the unedited codon (termed hydrophobicity), and considered information on the nucleotide immediately upstream (-1_nucleotide) and downstream (+1_nucleotide) of the candidate C.

In addition, we included two new measures, based on what we term the editing transition probability. We wanted to capture the fact that some codons are edited more frequently than others (originally observed in [10]), and that some amino acids are more frequently altered than others. To do so, we used transition probabilities, a standard measure of estimating the likelihood of finding a given codon or amino acid in a sequence [20]. In this case, we measured transition probabilities for both pre- and post-edited sequences. We derived these transition probabilities from the training data using maximum likelihood.

We chose a simple objective function that would assign a numeric value for each of the six variables based on the frequencies observed in the training data. In each iteration of cross-validation, these numeric values were calculated from the appropriate training data set and then applied to the testing data set. For example, in one training data set, 53.5% of the *A. thaliana *edit sites fall in the second codon position. Therefore, a putative edit site would receive a value of 0.535 for the variable codon_position if the cytosine under consideration was in the second codon position (see Additional File 2).

#### Defining the scoring function

Definition of the objective functions for the variables was simple and intuitive, but a method for combining these values into an overall score was not. The problem of combining the objective functions was formulated in terms of a linear scoring function for scoring putative editing sites. It was denned as:

*S*(*C*) = *W*_1 _*S*_1 _+ *W*_2 _*S*_2 _+ ...*W*_*n *_*S*_*n *_    (1)

where *S*(*C*) represents the score that would be assigned to a given cytosine based on its likelihood of being an edited site. *S*_*n *_corresponds to the nth objective function from one of the six variables and *W*_*n *_corresponds to an integer weight for that objective function. From this definition, the problem becomes one of function optimization: a need to define a set of weights that will most effectively separate the true positive and true negative groups. We utilized the GA to optimize this function.

#### Evaluating the fitness function

Definition of a fitness function for evaluating individual members of a population is perhaps the most crucial step in designing a genetic algorithm. In discriminating between edited and non-edited sites, the goal was to derive a fitness function that achieved maximal separation between the scores (derived from Equation 1) for edited and non-edited groups of cytosines. We utilized the following fitness function:

*F*(*O*) = *mean*(*S*(*C*_*E*_))/*mean*(*S*(*C*_*U*_))     (2)

where *F*(*O*) is the fitness value for a given organism, *S*(*C_E_*) would be the overall score for a given edited cytosine from Equation 1, and *S*(*C*_*U*_)would be the overall score for a given unedited cytosine. We take the mean score generated by an organism for all known edited cytosines in the training set and compare it to the mean score for all known unedited cytosines in the training set. The ratio of the mean scores provides a measure of the classification accuracy of a given organism. This fitness function rewards organisms that score edited sites with higher values than non-edited sites to encourage the development of effective classifiers.

#### Overview of implementation

There are many approaches to implementing GAs [16]. Our implementation is similar to other GAs applied to biological problems [21].

1. Genomes for all organisms in the population were initialized by setting the weights within Equation 1 to random values. There were 50 organisms per simulation.

2. Organisms were probabilistically selected for mutation and crossover in a manner proportional to their fitness. That is, highly successful organisms were selected for crossover and point mutations more often than less successful organisms.

3. Organisms were selected for death in a manner inversely proportional to their fitness. In other words, organisms that were poor classifiers were rapidly eliminated from the general population.

4. New organisms initialized as in Step 1 replaced any organisms killed in Step 3.

**Figure 2:**
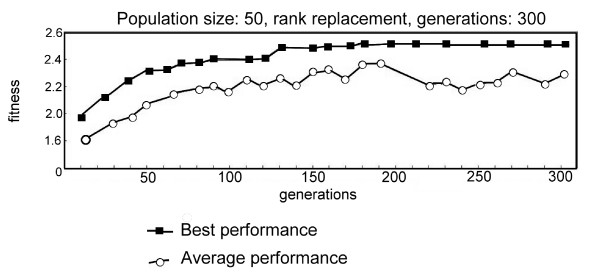
**Fitness of organisms over 300 generations**. We show the evolutionary progress of a population in REGAL. Each organism in the population represents a classifier for scoring edited and unedited cytosine nucleotides, and the fitness of any organism is given by the average score of the edited cytosines divided by the average score of the unedited cytosines. The line with circles represents the average performance of organisms within the simulation, and the line with black boxes represents the performance of the most fit organism within the simulation.

Steps 2–4 were repeated until algorithm termination. We ran the GA for 2,500 iterations in each simulation, and we had 100 simulations. The evolution of the GA over the first 300 generations in one of the simulations is shown in Figure 2. While some convergence was obtained as early as 300 generations, we noted additional improvement in later generations. We therefore allowed the GA to continue to evolve until no further improvement was noted.

The fundamental idea in a GA is that the fitness of an organism governs how likely its genetic material will be preserved in future generations. Three evolutionary operators were used to manipulate the fitness of the organisms: mutation, crossover and death. Mutation is used to introduce new genetic material into the simulation, so that the initial step of defining a population does not permanently limit the genetic material available. When using binary strings, a mutation operator simply selects a point along the chromosome at random. The value is flipped to zero if it were previously one, and to one if it were previously zero. In our implementation, one bit was flipped in each selected chromosome. All bits had an equal probability of being flipped.

We used single-point crossover, where a point is randomly selected along the chromosomes of two organisms. The chromosomes are split at this point, with the left hand portion of organism A's chromosome being swapped with the left hand portion of organism B's chromosome. By shuffling around bits of successful organisms within the population through crossover, this algorithm has a chance to try novel combinations of previously successful solutions.

Death is simply the removal of an organism and its genome from the population. In our implementation, the death of an organism triggers the initialization of a new organism to take its place. Thus, the population size remains stable throughout the simulation.

The fittest organisms from each generation and their associated fitness values were stored in a MySQL database for later examination. Code for the GA was implemented in the Perl programming language. Organisms were represented using a 96-bit binary genome, with 16 continuous binary numbers representing a single weight for a given variable.

### Developing REGAL

After training on the appropriate data, the best organism was selected and embodied as our RNA editing site predictor REGAL. The organism embodied within REGAL had the form:

001111000100111111100011101100101011110111110110111001001000011001111101101111011100000011100100

When run on genomic sequence, the output from REGAL is a score for each cytosine considered. This score is derived from the scoring function (Equation 1). We determined a threshold score for classifying known edited and unedited sites by evaluating scores in the training data sets. This threshold score would allow us to classify a given cytosine as edited (score greater than the threshold value) or unedited (score less than the threshold value). To identify the best threshold value, we evaluated the sensitivity and specificity of REGAL in the *A. thaliana *training data sets at a variety of threshold values. Threshold values were tested in increments of 100. Peak sensitivity and specificity were achieved at a mean threshold score value of 34173. Thus, in REGAL, any cytosine with a score of 34173 or greater would be predicted to be an edited site. Cytosines with scores less than 34173 would be marked as unedited sites. REGAL was evaluated by cross-validation on the *A. thaliana *mitochondrial genome and on the entire set of known edited sites and an equivalent number of randomly selected unedited sites from the *B. napus *and *O. sativa *genomes.

## Results

### Implementing a genetic algorithm for RNA editing

We wished to determine the relative importance of each of the six variables described in Implementation for accurately identifying cytosines that are edited. In GA parlance, the importance of a variable is captured by its weight. The set of optimized weights from the best performing organism is shown in Figure 1. The larger the numerical value of the weight, the greater its importance for accurate classification. As can be seen, the highest weight was assigned to amino acid transition probability, indicating that RNA editing yields a strong bias toward certain amino acids after editing compared with pre-edited transcripts. In addition, the position of the C with respect to the codon was also highly significant, as were the codon transition probability (likelihood that a given codon would be edited to another codon) and a preference for edits that yielded a more hydrophobic amino acid.

### Building REGAL: A predictor of RNA editing sites

Once we had optimized the weights for the variables based on the GA, we could incorporate them into a method for predicting which cytosines were most likely to be edited in a given sequence. We utilized the weights and the scoring function optimized by the GA to score each cytosine in our test data sets. To classify cytosines as likely edited or unedited, we next identified a threshold score of 34,173 that maximized accuracy (see Implementation). We evaluated REGAL's performance on three mitochondrial genomes: *A. thaliana, B. napus *and *O. sativa*. In the case of *A. thaliana*, we used cross-validation as described in Implementation. For the other two genomes, we utilized all the known edit sites and an equal number of randomly selected, known unedited sites. The results are summarized in Tables 1, 2 and 3. The overall accuracy is quite high, 87%. In particular, specificity, or the ability to eliminate non-edited sites, is consistently high at 91% for the three genomes. Sensitivity, or the ability to identify known edited sites is somewhat lower, ranging from 81% in the *B. napus *and *A. thaliana *genomes to 85% in the *O. sativa *genome. The complete predictions for all three genomes are provided in the accompanying files (see Additional Files 3, 4 and 5 respectively).

**Figure 3:**
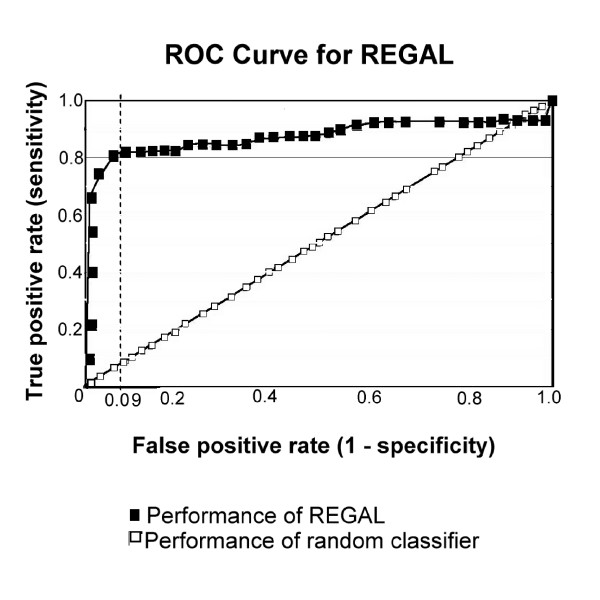
**ROC curve for REGAL**. ROC curves are used to evaluate the ability of a classifier to distinguish between true positives and false positives. With any classifier, increases in sensitivity will inevitably lead to more false positives. When a classifier behaves in an entirely random fashion, then each stepwise increase in sensitivity will lead to a stepwise increase in false positives. This is shown on the plot below as a 45° line. The better the classifier, the higher the curve rises along the left hand side of the plot, indicating stepwise increases in sensitivity with minimal increases in the false positive rate. This is the case for REGAL (line with black boxes). We note that the optimal point for sensitivity versus false positive rate is at a value of 0.82 for sensitivity and 0.09 for the false positive rate (indicated by the dashed line). In other words, the best performance by REGAL on the *A. thaliana *mitochondrial data set yields a sensitivity of 82% and a false positive rate of just 9%.

To assess the effectiveness of REGAL as a classifier, we generated a receiver operating characteristics (ROC) curve. ROC curves are used to measure the ability of a classifier to distinguish between true positives (known edited sites in this case) and false positives (known non-edited sites that are incorrectly predicted to be edited). Figure 3 shows the ROC curve for REGAL. As can be seen, the classifier is quite good, keeping the false positive rate low even while ensuring that most true positives are correctly identified. These results suggest that REGAL is a robust predictor of *C *→ *U *editing in mitochondrial genomic sequences.

### Comparing REGAL to other methods

The results of our approach compare favorably with the other *ab initio *method for RNA editing prediction [7]. That study utilized both classification trees and random forests that analyzed codon position, the nucleotides in a 40-base pair window around each cytosine, and the folding energy of the transcribed mRNA within the same window. Performance measures for this study are shown in Tables 4 and 5. The overall accuracy of classification trees is 70% compared with 87% for REGAL. In addition, both sensitivity and specificity are much higher for REGAL (64% versus 82% and 88% versus 91% respectively). Similarly, REGAL outperforms random forest trees, which have a reported accuracy of 84% compared to REGAL's 87%. The sensitivity of REGAL is much higher (71% for random forests versus 82% for REGAL) as is specificity (81% versus 91%).

Comparing the performance of REGAL to the sequence homology based approach, PREP-Mt, was more difficult. Before we could compare our performance to PREP-Mt, we needed to address what we perceive to be a serious concern in the reported results for PREP-Mt. When evaluating the accuracy of PREP-Mt, the author compared the small set of known edited sites against the entire set of all known, unedited cytosines. Accuracy calculated under these circumstances will yield uninformative, skewed values. [22].

In order to explain our concerns, we must digress momentarily to discuss how performance measures are calculated. Three measures are commonly used: sensitivity, specificity and accuracy. Sensitivity, or the ability to identify known edited sites, is calculated as *TP/*(*TP *+ *FN*) where TP are true positives and FN are false negatives. In this instance, true positives are known edited sites that are predicted to be edited sites. False negatives are known edited sites that are predicted to be unedited (see also Tables 1, 2, 3). Specificity is usually calculated as *TN/*(*TN *+ *FP*) where TN are true negatives and FP are false positives. In this case, true negatives are known unedited sites that are predicted to be unedited, and false positives are known unedited sites that are predicted to be edited. As might be expected, when a large number of true negatives are compared to a small number of true positives, even a very poor classifier will have a high accuracy. This is because proportionately fewer true negatives need to be identified correctly to achieve the same level of performance [23]. The author of PREP-Mt attempts to address this by using what he terms the "balanced accuracy," which he calculates as the mean of sensitivity and specificity. Given the potential skew in specificity values, simply averaging the specificity with sensitivity does not address the problem. In these circumstances, the correct approach is to use the positive predictive value (PPV) rather than specificity as in calculating accuracy. PPV is calculated as *TP/*(*TP *+ *FP*) [22,23].

For us to make a reasonable comparison of accuracy between PREP-Mt and REGAL, we had to re-calculate both the specificity and accuracy based on the raw numbers reported for PREP-Mt. To address the skew in specificity, we have calculated the PPVs for each of the three genomes shared in common between the PREP-Mt analysis and our approach. These values are shown in Table 6. Based on the PPV values, we also re-calculated accuracy as the mean of sensitivity and PPV. We used the reported sensitivity values for all edit sites predicted by PREP-Mt, including silent edit sites.

Overall, the performance of REGAL improves upon that of PREP-Mt. REGAL has a much higher specificity (91% for REGAL compared to 86% for PREP-Mt) and a comparable sensitivity (82% for both REGAL and PREP-Mt). REGAL has a higher accuracy (87% compared to PREP-Mt's 84%). The higher accuracy of REGAL is likely a result of its greater specificity in these data sets. In only one genome, *B. napus*, does REGAL have lower sensitivity than PREP-Mt. This leads to a slightly lower accuracy for REGAL (86% compared to 87% for PREP-Mt) in this genome. However, we note that the *B. napus *genome presents some challenges for all computational methods, in part because of uncertain annotations for some coding sequences and edit sites [7,8]. Therefore, the lower sensitivity may be a consequence of the data set rather than a direct reflection of REGAL's performance in this instance.

These results demonstrate the value of an *ab initio *method that utilizes a non-linear approach in prediction of *C *→ *U *RNA editing sites. Our accuracy, sensitivity and specificity are higher than other *ab initio *approaches, and REGAL has comparable sensitivity and higher specificity than sequence homology based methods. Moreover, there are certain general advantages to our approach which we outline in the following section.

## Discussion

The computational prediction of *C *→ *U *RNA editing sites has recently become feasible with the release of data sets such as the set of known editing sites in plant mitochondria [7,8]. At first glance, it might seem redundant to develop yet another method for the prediction of RNA editing sites in plant mitochondria, given the previous efforts in this field. However, our objective was to demonstrate the feasibility of our approach to the general challenge of predicting *C *→ *U *RNA editing sites. In this context, the plant mitochondrial genome data represent the best data sets for the development and testing of computational methods.

Here, we demonstrate the advantages of an *ab initio *approach that utilizes a non-linear, combinatorial method to identify known edit sites. REGAL, our predictive method derived from a genetic algorithm, is 87% accurate, with a sensitivity of 82% and a specificity of 91%. REGAL significantly outperforms the other *ab initio *approach, and it is more specific and more accurate than the sequence homology approach. This is especially striking since *ab initio *approaches generally tend to have lower specificity (i.e. more false positives) than sequence homology based approaches. As a result, our work demonstrates not only the feasibility of the approach, but also improves upon existing methods for identifying edit sites in plant mitochondrial genomes.

There are two advantages to an *ab initio *approach compared with sequence homology approaches. As mentioned earlier, sequence homology approaches rely on strong conservation of sequences. When such conservation is weak or non-existent, these methods fail to reliably predict edit sites. For example, when we consider PREP-Mt predictions on a per gene basis, sensitivity (ability to identify known edit sites) has an astonishing range: 25% to 95% depending on the gene considered [8]. Such variability limits the applicability of the method in systems where prior knowledge of editing sites is limited. In contrast, REGAL has a consistently high sensitivity (data not shown).

Secondly, an *ab initio *approach can identify edit sites that effect changes in the downstream protein product as well as silent edits. Silent edits in mRNAs may not have much impact on the downstream protein and are therefore difficult to predict by a sequence conservation method [8]. It is possible that silent editing nevertheless plays a critical role. A related phenomenon, synonymous substitutions across mammalian lineages, has been shown to have a dramatic impact on mRNA stability [24]. Given this finding, it is possible that silent editing serves a similar purpose in some mRNA transcripts. A method for identifying all RNA editing sites in a genome regardless of impact on downstream protein products is therefore highly desirable. As our results suggest, REGAL is a reliable predictor of all known edit sites in a given genome.

From a computational perspective, reliably predicting RNA editing sites seems to require more information than is present solely in the mRNA sequence. This would explain the relatively weaker performance of the other *ab initio *method, which focused exclusively on mRNA signals. Sequence homology approaches ignore all evidence at the mRNA sequence level, with the consequence that performance depends heavily on conservation across proteins. Our approach combines information from both the mRNA and downstream protein sequence, and we believe this contributes to its overall performance. Three of the four variables with the greatest weights in our algorithm are essentially variant measures of the same phenomenon: the impact of editing on the downstream protein sequence (Figure 1). The second highest weighted variable, however, is codon position. This is derived from the mRNA sequence. Thus our method is a synthesis of the two previous approaches and appears to benefit from the inclusion of information from both the mRNA and the protein sequence levels.

Furthermore, the GA approach we have developed can be used to investigate the nature of RNA editing in plant mitochondrial genomes. That is, we can consider the highest weighted variables and investigate these features in more detail. For example, our approach re-asserts the importance of codon position in selecting the edit site [7,10]. It is perhaps not surprising that the second codon position is preferentially edited over the other two positions. If the end result of RNA editing is to alter amino acid residues to yield a functional protein from an otherwise incorrect or damaged transcript [5,6,11], then editing the second codon position is most likely to yield this result. In the standard and mitochondrial genetic codes, the second codon position is nondegenerate for all codons (based on codon tables from the National Center for Biotechnology Information, NCBI). In other words, any change to the nucleotide in the second codon position will always yield a different amino acid. Thus, the most efficient solution for altering the composition of the protein product would be to edit the second codon position.

In contrast to the earlier *ab initio *approach, however, we were able to obtain good performance without the use of mRNA stability measures such as secondary structure. While Cummings and Myers reported significantly improved performance after incorporating free energy calculations into their algorithm [7], there does not appear to be much experimental evidence for the role of mRNA secondary structure in the selection of *C *→ *U *edit sites [25,26]. Given this experimental evidence, we decided to model edit sites without mRNA structural features. The fact that REGAL outperforms the Cummings and Myers approach without secondary structure features is in keeping with the experimental evidence in this regard. Nevertheless, it is possible that some aspect of mRNA secondary structure is involved in edit site selection. This is certainly an area for further investigation, both at the computational and experimental levels.

One of the most intriguing findings of our analysis is that the hydrophobicity of the edited amino acid tends to be higher than its pre-edited counterpart. This finding was noted in the original work detailing edit sites in the mitochondria of *A. thaliana *[10], but to our knowledge has not been investigated further. Why might hydrophobic residues be critical to the functionality of a protein? While we cannot speculate on specifics, we do note a recent study which demonstrates that increasing the number of hydrophobic residues can at times improve the stability of a protein [27]. Highly speculative as this is, it does inspire some intriguing questions with regard to the exact role of RNA editing in cellular systems.

As a general approach, the use of GAs to identify edit sites should be extensible to many other systems where *C *→ *U *RNA editing appears to play a role in gene expression. A primary constraint until recently has been identifying a sufficiently large data set of putative edit sites. Such a data set would serve two important purposes. First, it would provide a basis for developing the set of features required to identify edit sites. Second, sufficient data is required for adequate training and testing of an approach such as the one we describe here. The recent release of a comprehensive analysis of putative edit sites in the human transcriptome [28], for example, includes a large enough data set to both identify a set of likely features and train and test a method such as ours. We would suggest that the use of *ab initio*, nonlinear approaches could bring significant power to bear on the challenge of identifying *C *→ *U *RNA editing sites in genomes across the eukaryotic spectrum.

## Conclusion

REGAL is a robust classifier of *C *→ *U *RNA editing sites in plant mitochondrial genomes. Our method is quite accurate (87%) with high specificity (91%) and sensitivity (82%) across all three mitochondrial genomes tested. REGAL outperforms previous attempts at an *ab initio *approach and has comparable sensitivity and higher specificity than the sequence homology based approach. We believe REGAL's improved performance compared with previous efforts is a consequence of combining information from the mRNA and protein sequence levels. We would suggest that REGAL can be extended to other systems given some knowledge of the features of editing sites and sufficient training and testing data.

## Availability and requirements

REGAL is a collection of Perl scripts freely available under the Gnu General Public License (GPL). There are no restrictions on its use by non-academics. It has been tested on the Unix platform but may be extensible to other operating systems. REGAL requires several Perl modules and a MySQL database to store successful organisms during GA training. The full set of module and other requirements are listed in the user guide included with the distribution. The home page for REGAL is available online [29].

## Authors' contributions

JT designed and implemented the GA and developed the software required for REGAL. SG conceived of and guided the design of the GA and REGAL. Both authors contributed to the writing of this manuscript.

## Supplementary Material

Additional File 1 **Parsed CDS entries**. The final set of edit sites extracted from each of the mitochondrial genomes are included in the format of modified GenBank files. For each entry, we include the complete CDS and note the locations of edit sites within the CDS sequence. Click here for file

Additional File 2 **Objective function values obtained from *A. thaliana***. The set of values for each of the six variables utilized in the GA are reported here. These values are derived from the observed frequencies in the training data from *A. thaliana.* Click here for file

Additional File 3 **Objective scores and predictions for *A. thaliana***. The full set of known edit sites and known unedited sites used in one iteration of the cross-validation test data set from *A. thaliana *are included here. The values for each of the six variables, the overall score for each edit site and the REGAL prediction are listed. Click here for file

Additional File 4 **Objective scores and predictions for *B. napus***. Similar to the previous file, this includes all the variable values and predictions for the set of known edited and unedited sites in the *B. napus *genome. Click here for file

Additional File 5 **Objective scores and predictions for *O. sativa***. The equivalent file containing the set of variable values and predictions for the set of known edited and unedited sites in the *O. sativa *genome. Click here for file

## Figures and Tables

**Table 1:** Overall performance of REGAL on *A. thaliana*. We tested the performance of REGAL on known edited and unedited sites from three mitochondrial genomes. The results from *A. thaliana *were obtained after 100 iterations of cross-validation using 100 edited and 100 unedited sites per testing data set. We report the range of values as obtained from the cross-validation for this genome.

	**Known Edited Sites **Total: 100	**Known Unedited Sites **Total: 100	
**Predicted Edited Site**	True positive 81 (± 2.4)	False positive 9 (± 0.4)	**Sensitivity: 0.81 **(0.80–0.82) **Specificity: 0.91 **(0.90–0.92)
**Predicted Unedited Site**	False negative 19 (± 0.5)	True negative 91 (± 5.4)	**Accuracy: 0.86 **(0.86–0.90)

**Table 2:** Overall Performance of REGAL on *B. napus*. The performance of REGAL on the *B. napus *mitochondrial genome is shown here. REGAL was tested on 416 known edited sites and an equivalent number of known unedited sites.

	**Known Edited Sites **Total: 416	**Known Unedited Sites **Total: 416	
**Predicted Edited Site**	True positive 335	False positive 38	**Sensitivity: 0.81** **Specificity: 0.91**
**Predicted Unedited Site**	False negative 81	True negative 378	**Accuracy: 0.86**

**Table 3:** Overall Performance of REGAL on *O. sativa*. For the *O. sativa *mitochondrial genome, we tested REGAL on 481 known edited sites and 481 randomly selected, unedited sites.

	**Known Edited Sites **Total: 481	**Known Unedited Sites **Total: 481	
**Predicted Edited Site**	True positive 407	False positive 41	**Sensitivity: 0.85** **Specificity: 0.92**
**Predicted Unedited Site**	False negative 74	True negative 440	**Accuracy: 0.88**

**Table 4:** Comparison of REGAL vs. Classification Trees. Performance measures for predicting RNA editing were compared to the results as reported for classification trees [7]. REGAL outperforms classification trees in all three mitochondrial genomes.

	**Classification Trees**			**REGAL**		
	
	Sensitivity	Specificity	Accuracy	Sensitivity	Specificity	Accuracy
*A. thaliana*	0.65	0.89	0.71	0.81	0.91	0.86
*B. napus*	0.63	0.89	0.69	0.81	0.91	0.86
*O. sativa*	0.64	0.88	0.71	0.85	0.92	0.88

**Overall**	**0.64**	**0.88**	**0.70**	**0.82**	**0.91**	**0.87**

**Table 5:** Comparison of REGAL vs. Random Forests. REGAL also outperforms a second technique from [7] using random forest trees for the identification of *C *→ *U *editing sites in mitochondrial genomes.

	**Random Forests**			**REGAL**		
	
	Sensitivity	Specificity	Accuracy	Sensitivity	Specificity	Accuracy
*A. thaliana*	0.70	0.81	0.74	0.81	0.91	0.86
*B. napus*	0.73	0.81	0.77	0.81	0.91	0.86
*O. sativa*	0.72	0.81	0.72	0.85	0.92	0.88

**Overall**	**0.71**	**0.81**	**0.84**	**0.82**	**0.91**	**0.87**

**Table 6:** Comparison of REGAL vs. PREP-Mt. To compare performance between REGAL and PREP-Mt [8], we had to recalculate the reported values for specificity and accuracy as described in the text. We have compared performance for the three mitochondrial genomes that were shared in common between the PREP-Mt and REGAL analyses.

	**PREP-Mt**			**REGAL**		
	
	Sensitivity	Positive Predictive Value	Accuracy	Sensitivity	Specificity	Accuracy
*A. thaliana*	0.79	0.86	0.82	0.81	0.91	0.86
*B. napus*	0.87	0.87	0.87	0.81	0.91	0.86
*O. sativa*	0.81	0.85	0.83	0.85	0.92	0.88

**Overall**	**0.82**	**0.86**	**0.84**	**0.82**	**0.91**	**0.87**
