# Correction: genetic algorithm learning as a robust approach to RNA editing site site prediction

**DOI:** 10.1186/1471-2105-7-406

**Published:** 2006-09-06

**Authors:** James Thompson, Shuba Gopal

**Affiliations:** 1 Department of Biological Sciences, Rochester Institute of Technology, Rochester, NY 14623, USA

## Abstract

After the publication of [1], we were alerted to an error in our data. The error was an one-off miscalculation in the extraction of position information for our set of true negatives. Our data set should have used randomly selected non-edited cytosines (C) as true negatives, but the data generation phase resulted in a set of nucleotides that were each one nucleotide downstream of known, unedited cytosines. The consequences of this error are reflected in changes to our results, although the general conclusions presented in our original publication remain largely unchanged.

## Modifications to implementation

### Changes to data sets

After correcting for the one off error in the data generation phase, we re-evaluated the data sets for all three of the genomes analyzed. Since the publication of our original work, the mitochondrial genomes of all three species have been updated. We therefore decided to revise our data sets using the new (as of April 2006) GenBank files for *Arabidopsis thaliana, Brassica napus *and *Oryza sativa *([GenBank: NC_001284, GenBank: AP006644, GenBank: BA000029]).

As before, we focused on those edit sites associated with coding regions. In reviewing these updated GenBank files, we determined certain edit sites that were ambiguous for one of three reasons. Some *C *→ *U *editing sites could not be reliably assigned to one coding region, while others were not on the correct strand as the annotated coding region. A smaller proportion of annotated edit sites were not cytosines (C) in the genomic sequence on the strand containing the relevant coding region. In addition, a few coding regions involved complex processes such as *trans*-splicing, and the annotated CDS coordinates did not yield a coding sequence that could be translated to the reported protein sequence. These discrepancies were of some concern to us since we could not independently confirm the presence or absence of editing. We therefore chose to select a subset of edit sites from the annotated set that were unambiguous and could be reliably assigned to a coding region whose translation exactly matched the annotated entry. From the set of 455 annotated edit sites in the *A. thaliana *mitochondrial genome, we retained 344 edit sites as unambiguous (see Additional File 1). For the *B. napus *genome, we retained 397 edit sites out of 428 annotated sites (see Additional File 2), and in the *O. sativa *genome, we utilized 419 edit sites out of the 485 annotated sites (see Additional File 3). For each set of true positives selected from the annotated edit sites, we chose an equivalent number of true negatives after correcting for the one off error.

As before, we used the set of true positives and negatives from *A. thaliana *to train our genetic algorithm (GA) and tested its performance using cross validation. We made one minor change to the method of cross-validation, using 10-fold cross-validation. This process involves reserving a randomly selected 10% of the known edited and unedited sites for testing. The remaining 90% of the data are used for training the GA. Ten such iterative splits are conducted, with training and testing occurring after each split. This has been demonstrated to reliably sample the entire data space in a data set of this size [2]. The results reported are the average of performance across all ten iterative splits.

### Changes to GA development and training

In the process of reviewing our results with the corrected data, we had to modify our fitness function to improve performance. Our new fitness function is derived from the effect size statistic (also known as Cohen's *d'*), a measure of how far apart the means of two distributions are [3]. In this instance, the two distributions represent the GA scores for known true positives and known true negatives respectively (Figure 1). By using the effect size statistic, we could maximize the distance between these two distributions' means. In other words, we could obtain the best classification by ensuring that the means of the two distributions were as far apart as possible. The effect size statistic is calculated as follows:

$F(0)=\frac{\left(mean(S(C_{E}))-mean(S(C_{U}))\right)}{(\sigma (C_{E})+\sigma (C_{U}))/2}\;\left(1\right)$

where *F*(0) is the fitness value for a given GA organism, *S*(*C*_*E*_) is the overall score for a given edited cytosine (as obtained by the scoring function, see [1]) and *S*(*C*_*U*_) is the overall score for a given unedited cytosine. The denominator is the mean of the standard deviations for edited cytosines (σ(*C*_*E*_)) and unedited cytosines (σ(*C*_*U*_)). This fitness function provided a better measure of the performance of a given GA organism within the GA than the original fitness function described in [1].

**Figure 1:**
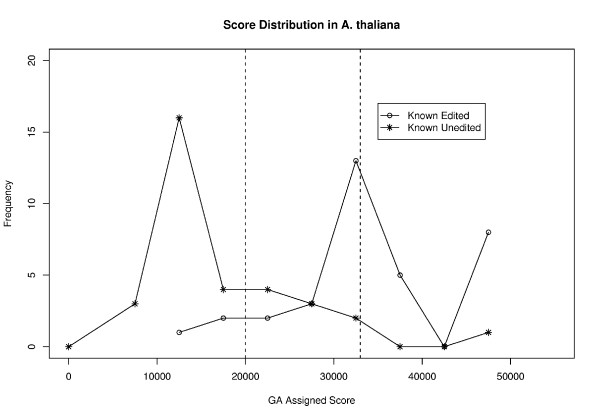
**Distribution of GA assigned scores for a test data set in *A. thaliana***. The distribution of GA assigned scores for one of the cross validation test data sets is shown here. This data set, which is fairly representative, had a total of 35 known edited sites and 37 known unedited sites, of which 23 known edited sites and 25 known unedited sites were in the 90% credible intervals. The dashed lines indicate the boundaries of the 90% credible intervals; a score of 20,000 or less indicates a ≥ 0.9 probability that the site is unedited and a score of 33,000 or greater indicates a ≥ 0.9 probability that a site is edited.

The objective values for each of the six variables remain as before (see Additional File 4).

Based on this new fitness function, we identified the best organism during 10-fold cross validation on the *A. thaliana *genome. This GA organism has a GA genome with the following structure:

010100111110111101101100100010001000111101111000110010101001010000100101000011100100011000000001

The above GA organism is now encoded in the updated version of REGAL (RNA Editing site prediction by Genetic Algorithm Learning) included here (see Additional File 5).

### Changes to REGAL output

In the course of reviewing our analysis, one aspect of the assessment of performance seemed to be somewhat limited in applicability. In our assessment of performance [1], we used sensitivity and specificity to demonstrate the ability of our classifier to make reliable predictions. That analysis provided an overall measure of the likelihood that predictions are correct. However, we did not assign an individual likelihood to each prediction so that users might immediately assess the likelihood that any given prediction is correct. We have now added an additional feature to the REGAL software that allows for an estimate of the likelihood that any given prediction is correct.

To implement this feature, we utilized the scores assigned to each known edited and unedited cytosine in the training data. We stepped through the scores in increments of 1000 asking at each step how many false positives would occur at that score level. We then identified a score level at which the false positive rate is as low as possible (see Additional File 4). In Figure 2, a score of 33,000 yields a false positive rate of just 10%. In other words, the likelihood that a cytosine with at least this score is edited is 90%.

**Figure 2:**
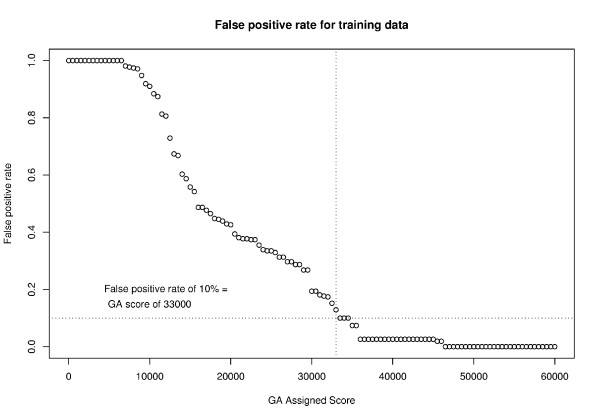
**GA score required for 0.9 or greater likelihood that a cytosine is edited**. We use the false positive rate to estimate the likelihood (posterior probability) that a given cytosine predicted to be edited is in fact edited. At a false positive rate of 10%, the posterior probability that a predicted edit site is a true edit site is 0.9. This corresponds to a GA assigned score of 33,000 or higher as shown in this plot.

Similarly we evaluated the range of scores and false negative rate at each level. The false negative rate at a given score level provides information on the likelihood that a prediction at that score is an unedited site. Figure 3 indicates that a false negative rate of 10% occurs at a score of 20,000 or less. That is, a cytosine scoring 20,000 or less would have a 90% likelihood of being unedited (see Additional File 4).

**Figure 3:**
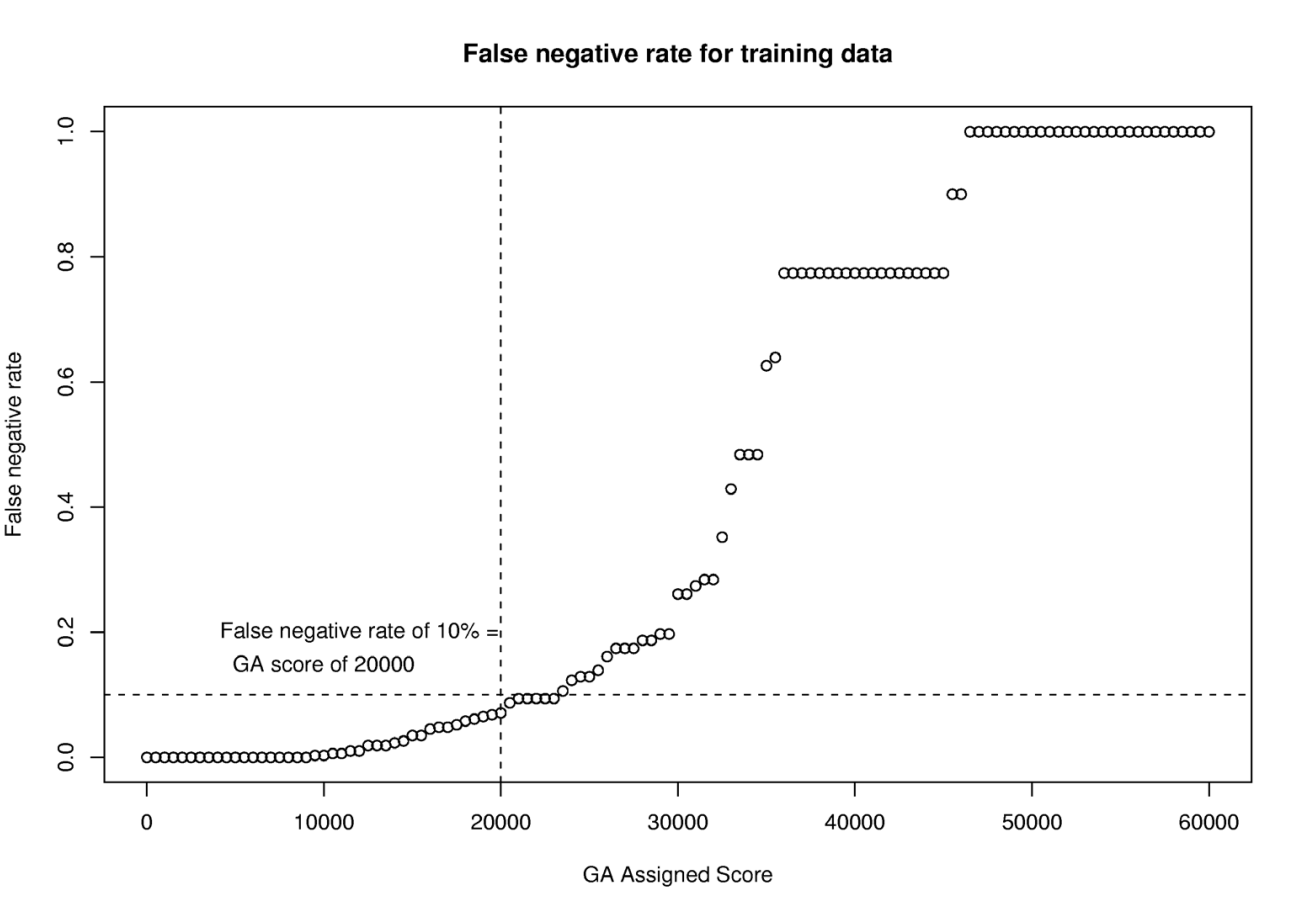
**GA score required for 0.9 or greater likelihood that a cytosine is unedited**. Similar to Figure 2, we use the false negative rate to estimate the posterior probability that a given cytosine will be unedited. The false negative rate of 10% corresponds to a GA assigned score of 20,000. Therefore, any cytosine with a score of 20,000 or less will have a 0.9 or greater likelihood of being unedited.

Since our analysis relies on Bayesian probability, these are the 90% credible intervals [4]. We can interpret these as roughly similar to the 90% confidence levels in a frequentist statistical analysis [5,6]. In other words, when REGAL predicts that a site is edited, and the score assigned to that site is greater than 33,000, we have at least 90% confidence that the prediction is true. Similarly, if REGAL were to assign a score less than 20,000 for a cytosine, we would have 90% or greater confidence that the site was unedited. In considering the performance of REGAL with the other methods for predicting edit sites in these genomes, we consider only those predictions that are in the 90% credible interval range. Considering results from a set of credible intervals is a well-established and accepted practice in the statistical analysis of classifiers [2, 5, ?, 7]. It allows us to assess the performance of REGAL based on those predictions that have the greatest confidence.

## Corrected results

The best performing organism generated by the GA has been encoded as REGAL (RNA Editing site prediction by Genetic Algorithm Learning), our method for predicting *C *→ *U *edit sites in plant mitochondrial genomes. The optimized weights for our six variables derived from this organism are shown in Figure 4. The larger the numerical value of the weight, the more important the variable is in classification of cytosines as edited or unedited. As before, the highest weight is assigned to amino acid transition probability, supporting our earlier conclusion that a certain bias seems to exist for the editing of some amino acids over others. In addition, the hydrophobicity of the amino acid continues to be a key indicator of the likelihood of editing. In contrast to our previous analysis, the nucleotides in the -1 and +1 positions now have higher weights, while codon position and codon transition probability are no longer significant contributors to accurate classification of sites.

**Figure 4:**
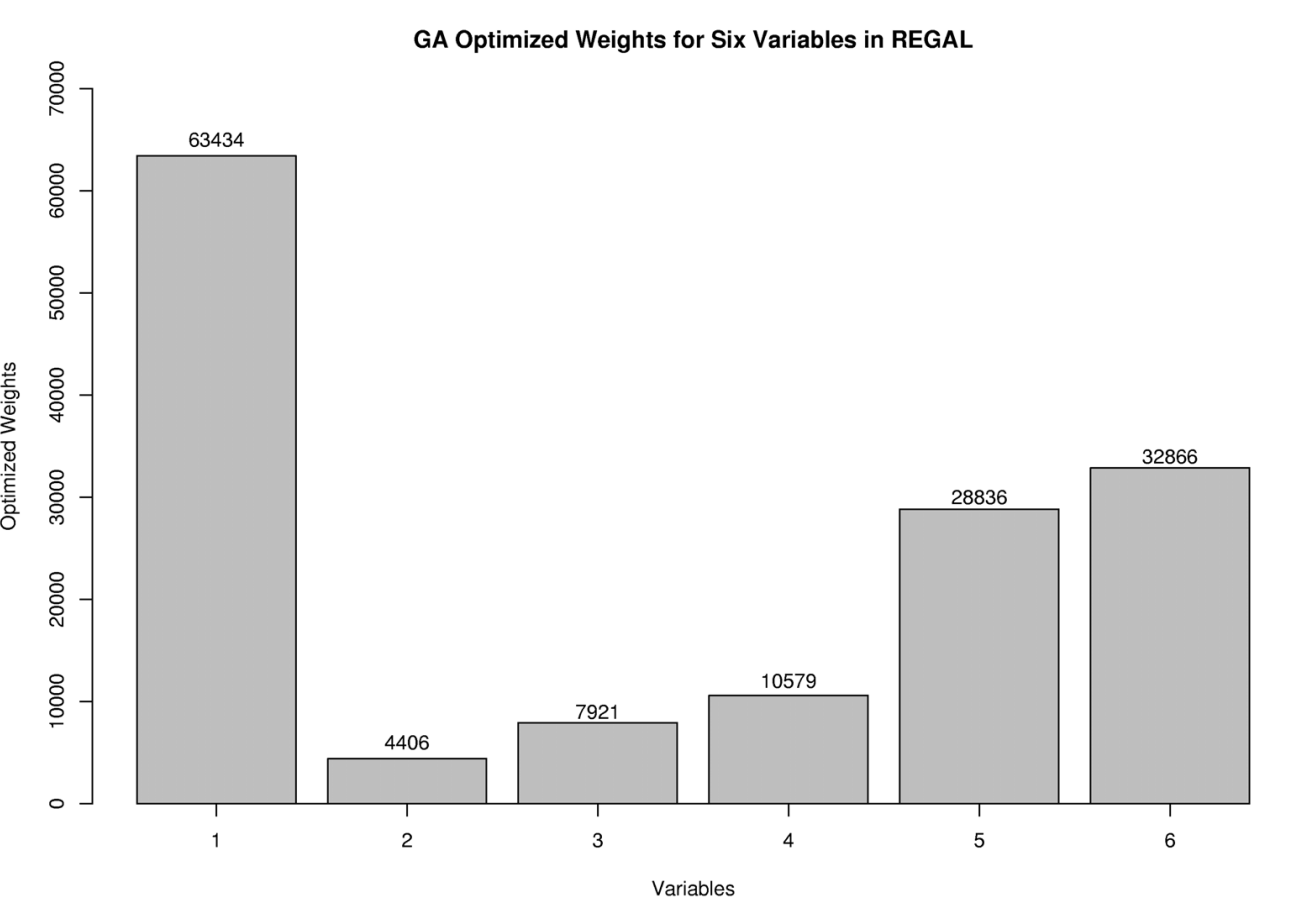
**GA optimized weights for six variables in REGAL**. We selected six variables and utilized the GA to optimize the weights for these variables (correction of Figure 1 from [1]). The greater the importance of a variable, the higher the value as shown here. Variables were abbreviated as follows: 1 = transition probability for amino acid pre- and post-edit; 2 = position of the candidate edit site within the codon; 3 = transition probability for codon pre- and post-edit; 4 = likelihood that editing will yield a more hydrophobic amino acid than the unedited codon; 5. = nucleotide in the -1 position; 6 = nucleotide in the +1 position.

Using the optimized weights, we scored each cytosine in the test data sets for *A. thaliana*, as well as the data sets from *B. napus *and *O. sativa*. REGAL now has an overall accuracy of 77%, with a sensitivity of 81% and a specificity of 74%. In the 90% credible interval range, the overall accuracy is 86%, with sensitivity of 89% and specificity of 83%. This is similar to our previously reported results, with sensitivity actually higher with the new organism. Specificity is somewhat reduced compared to our previously reported level. Nevertheless, the overall accuracy in the 90% credible intervals remains identical to our previous findings.

The output from REGAL now includes two values. The first is a score for a given cytosine assigned by the GA. Figure 1 shows the distribution of scores generated by REGAL for one of the test data sets from *A. thaliana*. The second output from REGAL is the posterior probability that the prediction is correct.

This value is estimated from the false positive and false negative rates, as described in Implementation. In Figure 1, the 90% credible intervals, based on this estimated posterior probability, are indicated by the dashed lines. In the subsequent description of results and in comparisons to other methods, we consider only the results from the 90% credible intervals. As discussed in Implementation, this is an accepted and well-established practice in evaluating the performance of classifiers [2, 5, ?, 7].

Figure 5 shows the ROC curve for REGAL when reporting sites in the 90% credible intervals. The ROC curve indicates that REGAL remains a good classifier of edit sites, since the curve is still well above what would be expected for a random classifier (shown in the dashed line).

**Figure 5:**
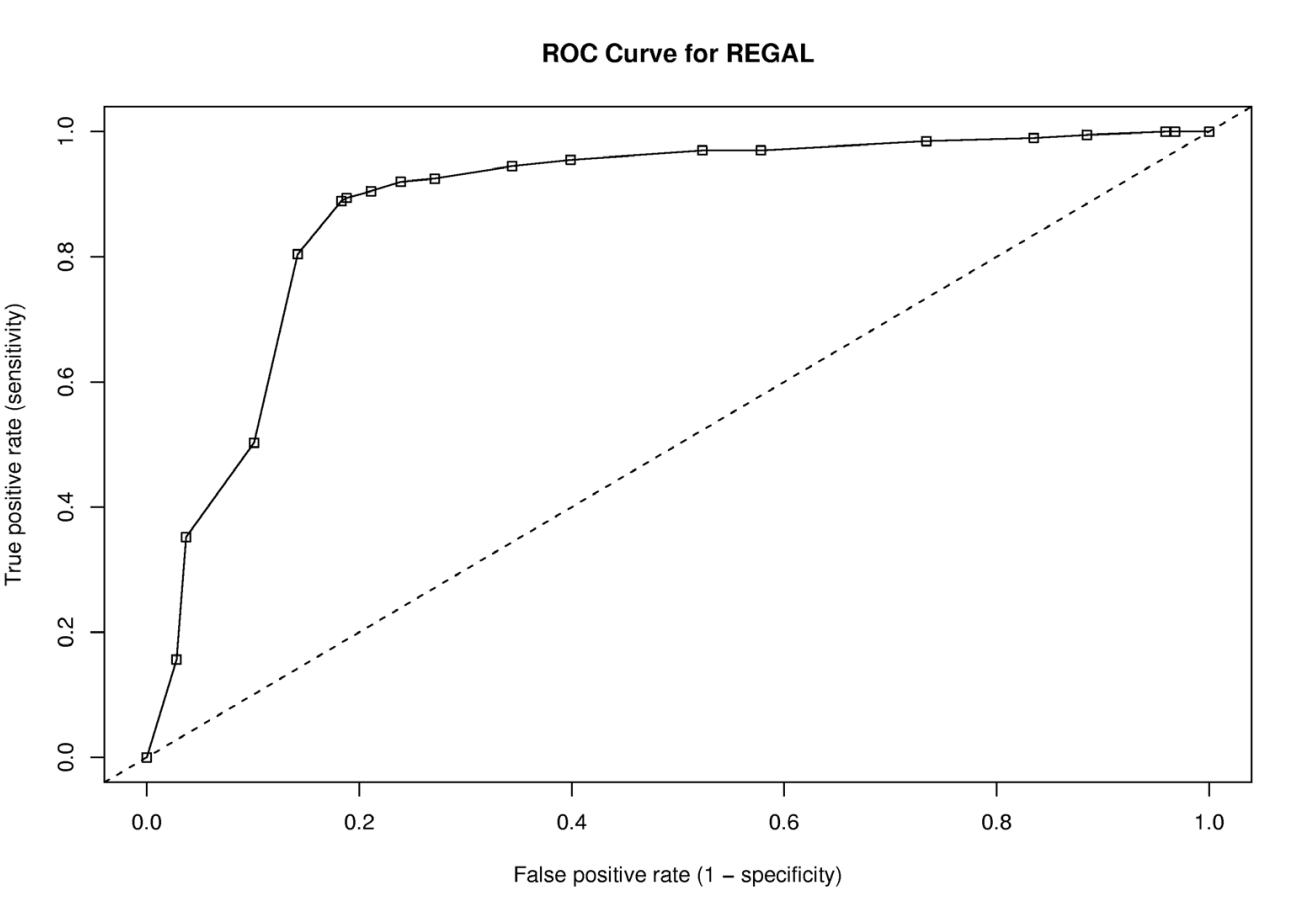
**ROC curve for REGAL**. The updated ROC curve for REGAL (correction from Figure 3 in [1]) is shown here. This represents the performance of the classifier as assessed using the 90% credible intervals as discussed in the text. The dashed line indicates performance of a random classifier. REGAL's performance is shown on the solid line with boxes.

In Tables 1, 2, 3, we report the corrected performance measures for REGAL for the three mitochondrial genomes analyzed, *A. thaliana, B. napus *and *O. sativa*. The new GA organism has much higher sensitivity across all three mitochondrial genomes than previously reported, and accuracy remains similar. Specificity (calculated as positive predictive value (PPV), see [1]) is somewhat reduced, as might be expected given the wider distribution of scores for known true negatives seen in Figure 1. The full set of predictions for each of the three genomes is included (see Additional Files 6, 7 and 8).

**Table 1:** Overall performance of REGAL on *A. thaliana*.

	Known Edited Sites Total: 17 – 26	Known Unedited Sites Total: 18 – 28	
**Predicted Edited Site**	True positive 19.4 (± 3.4)	False positive 3.3 (± 1.2)	**Sensitivity: 0.91 **(± 0.06) **Specificity: 0.85 **(± 0.06)
**Predicted Unedited Site**	False negative 2.0 (± 1.1)	True negative 19.7 (± 3.8)	**PPV: 0.86 **(± 0.05) **Accuracy: 0.88 **(± 0.05)

We tested the performance of REGAL on known edited and unedited sites from three mitochondrial genomes. The results from *A. thaliana *were obtained after 10 iterations of cross-validation using on average 33 edited and 33 unedited sites per testing data set (see Implementation for details of 10-fold cross-validation). The overall accuracy in this genome was 81%, with sensitivity of 81% and specificity of 80%. Within the 90% credible intervals, on average 22 edited sites and 23 unedited sites were assessed. We report the range of values as obtained from the cross-validation. Since the proportion of true positives to true negatives varied slightly in each test data set, we report both specificity and positive predictive value (PPV).

**Table 2:** Overall Performance of REGAL on *B. napus*.

	Known Edited Sites Total: 258	Known Unedited Sites Total: 263	
**Predicted Edited Site**	True positive 229	False positive 51	**Sensitivity: 0.89** **Specificity: 0.81**
**Predicted Unedited Site**	False negative 29	True negative 212	**PPV: 0.82** **Accuracy: 0.85**

The performance of REGAL on the *B. napus *mitochondrial genome is shown here. REGAL was tested on 397 known edited sites and an equivalent number of known unedited sites. The overall accuracy in this genome was 77%, with sensitivity of 83% and specificity of 72%. Of the full set of known edited and unedited sites, 258 known edited sites and 263 known unedited sites were in the 90% credible intervals. Because the numbers of true positives and true negatives are slightly different, PPV as well as specificity are shown.

**Table 3:** Overall Performance of REGAL on *O. sativa*.

	Known Edited Sites Total: 262	Known Unedited Sites Total: 287	
**Predicted Edited Site**	True positive 228	False positive 52	**Sensitivity: 0.87** **Specificity: 0.82**
**Predicted Unedited Site**	False negative 34	True negative 235	**PPV: 0.81** **Accuracy: 0.84**

For the *O. sativa *mitochondrial genome, we tested REGAL on 419 known edited sites and 419 randomly selected, unedited sites. The overall accuracy for this genome was 75%, with sensitivity of 79% and specificity of 71%. In the 90% credible intervals, there were 262 known edited sites and 287 known unedited sites. We report PPV as well as specificity.

### Comparing REGAL to other methods

We have updated Tables 4, 5, 6 to reflect our corrected results when comparing REGAL performance to the other methods for predicting edit sites in plant mitochondrial genomes. REGAL has a higher overall accuracy than the three other methods [8,9]. Of the methods available for analyzing these data, REGAL has the highest sensitivity (89%). In other words, REGAL is the best method to utilize to identify *C *→ *U *edit sites in these genomes. However, it may yield more false positives because the specificity (PPV) for REGAL is lower than for PREP-Mt [9], the next best method based on this assessment. The PPV difference between PREP-Mt (PPV of 86%) and REGAL (PPV of 83%) is relatively small. Furthermore, overall accuracy for REGAL (86%) is higher than for PREP-Mt (84%). As a result, we believe REGAL remains a valid alternative to the existing methods for predicting *C *→ *U *edit sites in plant mitochondrial genomes.

**Table 4:** Comparison of REGAL vs. Classification Trees.

	Classification Trees			REGAL		
	Sensitivity	Specificity	Accuracy	Sensitivity	Specificity (PPV)	Accuracy

*A. thaliana*	0.65	0.89	0.71	0.91	0.85 (0.86)	0.88
*B. napus*	0.63	0.89	0.69	0.89	0.81 (0.82)	0.85
*O. sativa*	0.64	0.88	0.71	0.87	0.82 (0.81)	0.84
**Overall**	**0.64**	**0.89**	**0.70**	**0.89**	**0.83 **(0.83)	**0.86**

Performance measures for predicting RNA editing were compared to the results as reported for classification trees [8]. We report both specificity and PPV (in parentheses after specificity values). REGAL has higher accuracy and sensitivity than classification trees in all three mitochondrial genomes.

**Table 5:** Comparison of REGAL vs. Random Forests.

	Random Forests			REGAL		
	Sensitivity	Specificity	Accuracy	Sensitivity	Specificity (PPV)	Accuracy

*A. thaliana*	0.70	0.81	0.74	0.91	0.85 (0.86)	0.88
*B. napus*	0.73	0.81	0.77	0.89	0.81 (0.82)	0.85
*O. sativa*	0.72	0.81	0.72	0.87	0.82 (0.81)	0.84
**Overall**	**0.72**	**0.81**	**0.74**	**0.89**	**0.83 **(0.83)	**0.86**

REGAL outperforms a second technique from [8] using random forest trees for the identification of *C *→ *U *editing sites in mitochondrial genomes. As before, we report PPV in parentheses.

**Table 6:** Comparison of REGAL vs. PREP-Mt.

	PREP-Mt			REGAL		
	Sensitivity	Positive Predictive Value	Accuracy	Sensitivity	Specificity (PPV)	Accuracy
*A. thaliana*	0.79	0.86	0.82	0.91	0.85 (0.86)	0.88
*B. napus*	0.87	0.87	0.87	0.89	0.81 (0.82)	0.85
*O. sativa*	0.81	0.85	0.83	0.87	0.82 (0.81)	0.84
**Overall**	**0.82**	**0.86**	**0.84**	**0.89**	**0.83 **(0.83)	**0.86**

To compare performance between REGAL and PREP-Mt [9], we had to recalculate the reported values for specificity and accuracy as described in [1]. We have compared performance for the three mitochondrial genomes that were shared in common between the PREP-Mt and REGAL analyses. We report both specificity and PPV (in parentheses) for our results.

We regret any inconvenience the error in the data generation phase may have caused. We wish to thank Jeffrey P. Mower for bringing this error to our attention, and Saria Awadalla for conducting an independent review of the software prior to publication of this correction.

## Supplementary Material

Additional File 1 ***A. thaliana *data file**. The set of edit sites and unedited sites with information for the six variables we used in training and testing are included in a tab separated file. Click here for file

Additional File 2 ***B. napus *data file**. The set of known edit sites and randomly selected unedited sites we utilized in this analysis along with the values for each of the six variables are listed in tab separated format. Click here for file

Additional File 3 ***O. sativa *data file**. The set of known edit sites and randomly selected unedited sites that we selected for this analysis are included, along with the values for the six variables. Click here for file

Additional File 4 **Objective function values obtained from *A. thaliana***. The set of values for each of the six variables utilized in the GA are reported here. These values are derived from the observed frequencies in the training data from *A. thaliana*. We also include the false positive and false negative rates for the range of GA scores from 0 to 60,000. These values are used in estimating the posterior probability that a given prediction in REGAL is correct. Click here for file

Additional File 5 **REGAL and scripts for GA evolution**. The complete set of scripts required for evolving, training and testing the GA and the implementation of the GA as REGAL are provided as a compressed tar archive. Click here for file

Additional File 6 **GA assigned scores and predictions for *A. thaliana***. The set of known edit sites and known unedited sites used in one iteration of testing from *A. thaliana *are included here. The overall score for each edit site, the estimated confidence in the prediction and the REGAL prediction are listed. Click here for file

Additional File 7 **GA assigned scores and predictions for *B. napus***. Similar to the previous file, this includes the overall scores, estimated confidence and predictions for the set of known edited and unedited sites in the *B. napus *genome. Click here for file

Additional File 8 **Objective scores and predictions for *O. sativa***. The equivalent file containing the set of overall scores, confidence estimates and predictions for the set of known edited and unedited sites in the *O. sativa *genome. Click here for file
