# Supplementary material for: Genetic algorithm learning as a robust approach to RNA editing site prediction
Source: BMC Bioinformatics. 2006 Mar 16;7:145. doi: 10.1186/1471-2105-7-145 (PMC1459874; doi:10.1186/1471-2105-7-145)
Supplement: Additional File 1 — Parsed CDS entries. The final set of edit sites extracted from each of the mitochondrial genomes are included in the format of modified GenBank files. For each entry, we include the complete CDS and note the locations of edit sites within the CDS sequence. [file 1471-2105-7-145-S1.pdf]

Values for GA Variables

We derived values for each of the variables utilized in our GA by estimating frequencies from the training data sets. These data were derived from the *A. thaliana* mitochondrial genome data. Each variable listed here corresponds to one of the six variables discussed in the text and shown in Figure 1.

Hydrophobicity values were based on the frequency with which an edited codon yielded a known hydrophobic amino acid. For example, 34% of all edited codons yielded a serine, so the value for serine would be 0.34 as shown below.

Hydrophobicity Values

Amino acid	Value
S	0.3411
P	0.2670
R	0.1505
L	0.0729
H	0.0565
I	0.0305
A	0.0117
V	0.0071
C	0.0024
Y	0.0024
Q	0.0024
D	0.0024

Amino acid transition probabilities were calculated based on the frequency of finding an amino acid in a transcript post-editing compared with pre-editing. The values below represent the frequency seen in the training data pre- and post-editing. Some amino acids were never edited in our data sets, and these have no entries in the post-editing portion of the table. Other amino acids were edited to more than one amino acid as shown by double entries below.

Amino Acid Transition Probabilities

Pre-edit Amino acid	Post-edit Amino acid	Transition Probability
A	A	0.00234741784037559
	V	0.0140845070422535
F	F	0.0234741784037559
S	S	0.0117370892018779
	F	0.115023474178404
	L	0.211267605633803
T	M	0.00469483568075117
	T	0.00704225352112676
	I	0.0164319248826291
P	F	0.0187793427230047
	S	0.0633802816901408
	P	0.00704225352112676
	L	0.199530516431925
Y	Y	0.00234741784037559
V	V	0.00704225352112676
H	Y	0.0539906103286385
Q	*	0.00469483568075117
D	D	0.00234741784037559
I	I	0.0211267605633803
R	W	0.0610328638497653
	C	0.0751173708920188
	R	0.00234741784037559
	*	0.00234741784037559
G	G	0.00234741784037559
L	F	0.0422535211267606
	L	0.028169014084507

Codon transition probabilities were estimated from the training data in a similar manner as the amino acid transition probabilities. These are listed below for each pre-edit codon and its corresponding post-edit versions.

Codon Transition Probabilities

Pre-edit codon	Post-edit codon	Transition Probability
GCC	GTT	0.00234741784037559
CTT	TTT	0.028169014084507
GGA	GGA	0.00469483568075117
GTC	GTC	0.00234741784037559
TCA	TTA	0.117370892018779
CGA	TGA	0.00234741784037559
ATT	ATT	0.00704225352112676
TAT	TAT	0.00234741784037559
TAC	TAC	0.00234741784037559
ACA	ATA	0.00469483568075117,
	ACA	0.00234741784037559
TCG	TTG	0.0985915492957746
ACT	ATT	0.00938967136150235
CAA	TAA	0.00234741784037559
CCG	TTG	0.0140845070422535,
	TCG	0.0117370892018779,
	CTG	0.0352112676056338
CTG	TTG	0.00469483568075117,
	CTG	0.00234741784037559
GGT	GGT	0.00704225352112676
GCA	GTA	0.00234741784037559
GTG	GTG	0.00234741784037559
TCC	TTT	0.00469483568075117,
	TTC	0.0328638497652582
TTT	TTT	0.00234741784037559
CAC	TAC	0.0140845070422535
CGT	CGT	0.00234741784037559,
	TGT	0.0586854460093897
CAT	CAT	0.00234741784037559,

	TAT	0.039906103286385
CGG	TGG	0.0610328638497653
ATA	ATA	0.00469483568075117
ACC	ACC	0.00234741784037559,
	ATC	0.00234741784037559
CCC	TCC	0.00704225352112676,
	CCC	0.00234741784037559,
	TCT	0.00234741784037559,
	TTC	0.00469483568075117,
	CTC	0.00938967136150235
TTA	TTA	0.00234741784037559
CCA	TCA	0.0117370892018779,
	CTA	0.068075117370892,
	TTA	0.0187793427230047
CTA	TTA	0.0140845070422535
GAT	GAT	0.00234741784037559
TCT	TTT	0.0751173708920188,
	TCT	0.00704225352112676
CTC	TTT	0.00469483568075117,
	CTT	0.00234741784037559,
	TTC	0.00938967136150235
CGC	TGC	0.0234741784037559
TTG	TTG	0.00234741784037559
GCG	GTG	0.00469483568075117
GGC	GGC	0.00234741784037559
GAA	GAA	0.00234741784037559
CAG	CAG	0.00234741784037559,
	TAG	0.00234741784037559
GCT	GCT	0.00234741784037559,
	GTT	0.00234741784037559
CCT	TTT	0.0140845070422535,
	CTT	0.0539906103286385,
	TCT	0.0328638497652582
ACG	ATG	0.00469483568075117
AAA	AAA	0.00469483568075117
ATG	ATG	0.00704225352112676
GTA	GTA	0.00234741784037559

The frequency of an edit occurring in each of the three codon positions is listed below.

Codon Position

First Position	Second Position	Third Position
0.349	0.535	0.116

The frequency with which each of the four nucleotides occur in the -1 position with respect to the edited C is listed below.

-1 Nucleotide

A	C	G	U
0.040	0.305	0.024	0.631

Nucleotide frequencies in the +1 position, immediately downstream of the edited C are listed here.

+1 Nucleotide

A	C	G	U
0.29	0.162	0.325	0.223
