# Supplementary material for: Correction: genetic algorithm learning as a robust approach to RNA editing site site prediction
Source: BMC Bioinformatics. 2006 Sep 6;7:406. doi: 10.1186/1471-2105-7-406 (PMC1569880; doi:10.1186/1471-2105-7-406)
Supplement: Additional File 5 — REGAL and scripts for GA evolution. The complete set of scripts required for evolving, training and testing the GA and the implementation of the GA as REGAL are provided as a compressed tar archive. [file 1471-2105-7-406-S5.gz › regal/user_guide.pdf]

REGAL: RNA Editing site prediction by Genetic Algorithm Learning

Shuba Gopal and James Thompson

Contents

1 **Overview of Software** **3**

2 **Using REGAL and the GA training software** **3**
2.1 The REGAL scripts: An overview 4
2.2 Other Requirements 5

3 **How to run REGAL: Flow of Scripts** **5**
3.1 Using REGAL with the existing organism 6
3.1.1 Running REGAL on a genome with prior knowledge of edit sites . . . 6
3.1.2 Running REGAL on a new genome with no prior edit information . . 6
3.2 Training the GA 7
3.3 Testing Organisms 7

4 **Questions and comments** **8**

5 **License** **9**

1 Overview of Software

REGAL, **R**NA **E**diting site prediction by **G**enetic **A**lgorithm **L**earning, is designed to predict $C \rightarrow U$ edit sites in plant mitochondrial genomes. REGAL is based on a genetic algorithm (GA), a machine learning method based on non-linear, combinatorial approaches to function optimization. There are two parts to this software: methods for developing and training the GA, and the REGAL software for predicting $C \rightarrow U$ edit sites in plant mitochondrial genomes.

Both the GA and REGAL were trained on the mitochondrial genome of *Arabidopsis thaliana*. The method's performance was assessed by cross-validation on this genome and independent testing on the *Brassica napus* and *Oryza sativa* mitochondrial genomes. The full details of the analysis of the method and its implementation are available in [1]. Additional details on the publication, as well as any updates to this software package, will be available on the home page for this project:

http://bioinformatics.rit.edu/~shuba

2 Using REGAL and the GA training software

We anticipate two kinds of users for this software. Some will wish to re-train the GA on a new genome, for which they will need access to the training methods and scripts. Others will wish to simply apply REGAL to a new genome without training. We have included relevant directions both for training the GA and for using REGAL on a genomic sequence.

REGAL is written in Perl, and the accompanying scripts are described briefly here and in more detail in the ensuing sections. The basic input into REGAL comprises a tab separated file of values for each of the six variables utilized by the GA. We no longer include data generation scripts in this software because GenBank files are very variable in format, and we cannot guarantee consistent results in parsing these files. The input for REGAL should be of the following format (resembling Additional Files 1-3 of [2]).

```
#ID      Position    CP  Minus 1 Plus 1 Pre-cod  Post-cod Pre-AA Post-AA Edit
atp1_1        1484   P1    C        A       CCA     CTA      P      L       +
atp1_2        1415   P0    A        G       CGA     TGA      P      *       +
```

Identifiers MUST be unique to each site (i.e. each site, regardless of whether it is edited or not must have its own identifier). Lines that are preceded with # will be ignored as comment lines. Codon position (CP) should be listed as P0 for first position, P1 for second position and P2 for third position. Minus 1 indicates the nucleotide in the -1 position, Plus 1 for the +1 nucleotide. Pre-cod is the codon prior to editing, and post-cod indicates the codon after editing. Similarly, Pre-AA and Post-AA refer to the amino acid prior to editing (Pre-AA) and post-editing (Post-AA). If the edit status of the site is known, indicate it as + (edited) or - (unedited). If the status is not known, randomly assign some sites to one status or the other. The order of values listed here must be preserved for correct parsing of the data files.

Following this, a script generates the training, testing and other data required for the GA and REGAL. These files can then be used to either train the GA or to generate predictions using REGAL. These scripts and their input and output are described in Section 2.1.

2.1 The REGAL scripts: An overview

The following scripts comprise the REGAL software package:

Script name	Description
**split_data.pl**	Script to generate data files<br>Input: File similar to Additional Files 1-3 in [2])<br>Output: training.data, testing.data and total.data<br>These files must not be renamed as<br>downstream scripts are hardcoded.
**evolve.pl**	Script to evolve a GA<br>Input: training.data and testing.data<br>Output: prints to screen top 10% of organisms with<br>fitness and other information.
**regal.pl**	Script to predict edit sites<br>Input: total.data and user specified values<br>Output:<br>regal_predictions_1.txt, a set of predictions<br>for the sites listed in total.data<br>performance_eval_1.txt, evaluation of a new<br>organism's performance if specified
**run_evolution.pl**	Script to automate the running of evolve.pl<br>Input: See options in usage statement<br>Output: Multiple runs of the GA with statistics<br>for use with cross-validation data sets.
**assess_org_perf.pl**	Script to assess the performance of<br>organisms generated by **run_evolution.pl**.<br>Input: See options in usage statement<br>Output: Summary statistics on the average accuracy<br>sensitivity and specificity of an<br>organism across cross-validation<br>data sets.

2.2 Other Requirements

The REGAL scripts utilize publicly available Perl modules for the evolution of the GA. These can be obtained through CPAN (http://www.cpan.org) The following are required for REGAL to operate:

1. **Perl**
   - REGAL does not require a specific version of Perl *per se*.
   - However, some of the modules it depends upon assume Perl 5.0 or higher. Check the relevant documentation for these modules for version requirements.
2. **REGAL specific Perl module**
   - There is a REGAL specific Perl module included in this distribution. This modules can either be installed to the general location of Perl modules on your system or it should be present in the same directory that contains the other scripts.

**EditPredictor.pm**	Set of methods specific to REGAL.<br>Includes methods both for training and testing GA<br>as well as methods for predicting edit sites.

3. **Other Perl modules** required by REGAL:
   - Algorithm::Evolve
   - Getopt::Long
   - GD::Graph::lines
   - File::Basename
   - Statistics::Descriptive::Discrete

3 How to run REGAL: Flow of Scripts

This software distribution includes scripts to train a GA, test it on a known genome with prior knowledge of edit sites and to predict edit sites in a new genome using either a pre-existing, trained organism or a new organism obtained by a fresh round of training and testing. As a result, the instructions that follow are designed to address the needs of all users and all permutations of options possible within the constraints of the code.

For any and all uses of this software the following two steps are generally required:

1. Generate an input data file as described earlier (similar to Additional Files 1-3 in [2]).

2. Use **split_data.pl** to generate training.data, testing.data and total.data files.

These data files should be stored in the same directory as the scripts to ensure easy access. It is best to run all the scripts from the same directory that contains EditPredictor.pm. Output is written to the local directory unless otherwise specified.

3.1 Using REGAL with the existing organism

To run REGAL on a new genome without training a new organism only one script, regal.pl, is required. There are two possible uses of REGAL in this mode: to assess its performance on a genome with prior knowledge of edit sites and to predict edit sites in a new genome without prior knowledge of edit sites.

3.1.1 Running REGAL on a genome with prior knowledge of edit sites

The following would be the input to regal.pl in this instance:

- `total.data` file (generated by make-data.pl)
- `-s predict` option at command line

The command to regal.pl would look something like this:

```
> regal.pl -s predict -out output.file
```

The -s flag indicates that regal.pl should be run to predict edit sites. The -out flag allows the user to specify an output file name. Otherwise, the default file names are utilized. The -s flag is required for regal.pl, but the -out flag is optional.

The output from regal.pl depends on the choice of -s option. When -s is set to "predict," the output is a file called regal_predictions_1.txt (unless the user specifies an output file name with the -out option). This plain text file contains an assessment of all the edit sites present in the total.data file. Each edit site is listed with its GA assigned score, estimated posterior probability and a prediction. If the edit status is known, that is also listed.

Here is an example of what the regal_predictions_1.txt file might look like:

```
#ID     Edit Score       Edit Confidence Edit Prediction Known Edit Status
atp1_1        34650.78        0.926           +          +
atp1_2        29731.97        0.806           +          -
```

3.1.2 Running REGAL on a new genome with no prior edit information

When running on a new genome where no prior knowledge of edit sites exists, all the putative cytosines can be listed in the total.data file. You will still need to generate the training.data and testing.data files using split_data.pl, but these files will not be utilized. However, their presence is required for proper running of the scripts.

Some of the methods that REGAL uses assume prior knowledge regarding edited sites. For a new genome, this can be simulated by randomly assigning some of the sites to the "+" state and some to the "-" state. The assignment will not affect the predictions. The call to REGAL would still be:

```
> regal.pl -s predict -out output.file
```

3.2 Training the GA

To train the GA on a new genome, begin by generating input data files similar to Additional Files 1-3 in [2]. Generate the training.data, testing.data and total.data files using split_data.pl. The next step is to run evolve.pl, which will generate statistics on the top 10% of best organisms after evolution is complete. When evolution has gone through the specified number of generations, it will end and print these statistics to the screen. Output can be redirected from the command line if desired.

To run more than one iteration of the GA, use run_evolution.pl. This will allow you to specify how many different instances of GA evolution you wish to run, as well as parameters for the GA evolution. You can assess the overall results of multiple GA runs using assess_org_perf.pl. These scripts assume you will be doing cross-validation on your data.

3.3 Testing Organisms

To test the performance of a single organism, use the regal.pl script (use assess_org_perf.pl for collective assessment of many organisms' performance). The options to regal.pl will be different from those when the existing organism are used, however. Here is a sample command line call when testing a new genome:

```
> regal.pl -s test -g 01011010111101100011010111100000010011110010
  -out output.file
```

where -s is set to the "test" state to indicate that you wish to assess the accuracy of a new organism. In addition, you may choose to specify an output file name using the -out flag. This is optional. The -g flag provides the full binary genome to use in scoring the sites.

More than one genome can be tested in one iteration of the script. That is, you can specify all the genomes of interest and obtain results for each of them. Simply specify each genome with its own -g flag when calling regal.pl.

The output from regal.pl in this instance will be the file performance_eval_1.txt (unless an output file name is specified with -out). For each genome, a separate file will be generated and labeled as performance_eval_1.txt, performance_eval_2.txt, etc. A sample performance_eval_1.txt file might look like this:

```
#Genome Structure:
#aa_transition => 63434
#codon_position => 4406
#codon_transition => 7921
#hydrophobicity => 10579
#minus_one_nuc => 28836
#plus_one_nuc => 32866
#Genome: 010100111110111101101100100010001000111110111
#Accuracy       Sensitivity     Specificity     Fitness
0.809   0.824   0.794   1.502
---------------------------------------------------------------------------
#ID     Edit Score      Edit Confidence Edit Prediction Known Edit Status
atp6-2   15527.2242197183    0.955            -             -
```

The binary genome is recoded into a decimal value for each of the six features, and then the fitness, accuracy and other measures are calculated and listed. Individual predictions for each site in testing.data are listed, along with the GA assigned score and the estimated posterior probability.

4 Questions and comments

This brief overview of the scripts should be sufficient for most users to apply REGAL to their specific context. Questions or comments on the usage, application or further development of this software are welcome! For further information or to clarify usage, please contact:

Shuba Gopal
Assistant Professor, Bioinformatics
Department of Biological Sciences
Rochester Institute of Technology
85 Lomb Memorial Drive
Rochester, NY 14623
Email: sxgsbi@rit.edu
Web: http://bioinformatics.rit.edu/~shuba

5 License

This software is available under the Gnu General Public License as described below.

GNU General Public License

Terms and Conditions For Copying, Distribution and Modification

0. This License applies to any program or other work which contains a notice placed by the copyright holder saying it may be distributed under the terms of this General Public License. The "Program", below, refers to any such program or work, and a "work based on the Program" means either the Program or any derivative work under copyright law: that is to say, a work containing the Program or a portion of it, either verbatim or with modifications and/or translated into another language. (Hereinafter, translation is included without limitation in the term "modification".) Each licensee is addressed as "you".

   Activities other than copying, distribution and modification are not covered by this License; they are outside its scope. The act of running the Program is not restricted, and the output from the Program is covered only if its contents constitute a work based on the Program (independent of having been made by running the Program). Whether that is true depends on what the Program does.

1. You may copy and distribute verbatim copies of the Program's source code as you receive it, in any medium, provided that you conspicuously and appropriately publish on each copy an appropriate copyright notice and disclaimer of warranty; keep intact all the notices that refer to this License and to the absence of any warranty; and give any other recipients of the Program a copy of this License along with the Program.

   You may charge a fee for the physical act of transferring a copy, and you may at your option offer warranty protection in exchange for a fee.

2. You may modify your copy or copies of the Program or any portion of it, thus forming a work based on the Program, and copy and distribute such modifications or work under the terms of Section 1 above, provided that you also meet all of these conditions:

   (a) You must cause the modified files to carry prominent notices stating that you changed the files and the date of any change.

   (b) You must cause any work that you distribute or publish, that in whole or in part contains or is derived from the Program or any part thereof, to be licensed as a whole at no charge to all third parties under the terms of this License.

   (c) If the modified program normally reads commands interactively when run, you must cause it, when started running for such interactive use in the most ordinary way, to print or display an announcement including an appropriate copyright notice and a notice that there is no warranty (or else, saying that you provide a warranty) and that users may redistribute the program under these conditions,

and telling the user how to view a copy of this License. (Exception: if the Program itself is interactive but does not normally print such an announcement, your work based on the Program is not required to print an announcement.)

These requirements apply to the modified work as a whole. If identifiable sections of that work are not derived from the Program, and can be reasonably considered independent and separate works in themselves, then this License, and its terms, do not apply to those sections when you distribute them as separate works. But when you distribute the same sections as part of a whole which is a work based on the Program, the distribution of the whole must be on the terms of this License, whose permissions for other licensees extend to the entire whole, and thus to each and every part regardless of who wrote it.

Thus, it is not the intent of this section to claim rights or contest your rights to work written entirely by you; rather, the intent is to exercise the right to control the distribution of derivative or collective works based on the Program.

In addition, mere aggregation of another work not based on the Program with the Program (or with a work based on the Program) on a volume of a storage or distribution medium does not bring the other work under the scope of this License.

3. You may copy and distribute the Program (or a work based on it, under Section 2) in object code or executable form under the terms of Sections 1 and 2 above provided that you also do one of the following:

   (a) Accompany it with the complete corresponding machine-readable source code, which must be distributed under the terms of Sections 1 and 2 above on a medium customarily used for software interchange; or,

   (b) Accompany it with a written offer, valid for at least three years, to give any third party, for a charge no more than your cost of physically performing source distribution, a complete machine-readable copy of the corresponding source code, to be distributed under the terms of Sections 1 and 2 above on a medium customarily used for software interchange; or,

   (c) Accompany it with the information you received as to the offer to distribute corresponding source code. (This alternative is allowed only for noncommercial distribution and only if you received the program in object code or executable form with such an offer, in accord with Subsection b above.)

   The source code for a work means the preferred form of the work for making modifications to it. For an executable work, complete source code means all the source code for all modules it contains, plus any associated interface definition files, plus the scripts used to control compilation and installation of the executable. However, as a special exception, the source code distributed need not include anything that is normally distributed (in either source or binary form) with the major components (compiler, kernel, and so on) of the operating system on which the executable runs, unless that component itself accompanies the executable.

If distribution of executable or object code is made by offering access to copy from a designated place, then offering equivalent access to copy the source code from the same place counts as distribution of the source code, even though third parties are not compelled to copy the source along with the object code.

4. You may not copy, modify, sublicense, or distribute the Program except as expressly provided under this License. Any attempt otherwise to copy, modify, sublicense or distribute the Program is void, and will automatically terminate your rights under this License. However, parties who have received copies, or rights, from you under this License will not have their licenses terminated so long as such parties remain in full compliance.

5. You are not required to accept this License, since you have not signed it. However, nothing else grants you permission to modify or distribute the Program or its derivative works. These actions are prohibited by law if you do not accept this License. Therefore, by modifying or distributing the Program (or any work based on the Program), you indicate your acceptance of this License to do so, and all its terms and conditions for copying, distributing or modifying the Program or works based on it.

6. Each time you redistribute the Program (or any work based on the Program), the recipient automatically receives a license from the original licensor to copy, distribute or modify the Program subject to these terms and conditions. You may not impose any further restrictions on the recipients' exercise of the rights granted herein. You are not responsible for enforcing compliance by third parties to this License.

7. If, as a consequence of a court judgment or allegation of patent infringement or for any other reason (not limited to patent issues), conditions are imposed on you (whether by court order, agreement or otherwise) that contradict the conditions of this License, they do not excuse you from the conditions of this License. If you cannot distribute so as to satisfy simultaneously your obligations under this License and any other pertinent obligations, then as a consequence you may not distribute the Program at all. For example, if a patent license would not permit royalty-free redistribution of the Program by all those who receive copies directly or indirectly through you, then the only way you could satisfy both it and this License would be to refrain entirely from distribution of the Program.

   If any portion of this section is held invalid or unenforceable under any particular circumstance, the balance of the section is intended to apply and the section as a whole is intended to apply in other circumstances.

   It is not the purpose of this section to induce you to infringe any patents or other property right claims or to contest validity of any such claims; this section has the sole purpose of protecting the integrity of the free software distribution system, which is implemented by public license practices. Many people have made generous contributions to the wide range of software distributed through that system in reliance on consistent application of that system; it is up to the author/donor to decide if he or she is willing to distribute software through any other system and a licensee cannot impose that choice.

This section is intended to make thoroughly clear what is believed to be a consequence of the rest of this License.

8. If the distribution and/or use of the Program is restricted in certain countries either by patents or by copyrighted interfaces, the original copyright holder who places the Program under this License may add an explicit geographical distribution limitation excluding those countries, so that distribution is permitted only in or among countries not thus excluded. In such case, this License incorporates the limitation as if written in the body of this License.

9. The Free Software Foundation may publish revised and/or new versions of the General Public License from time to time. Such new versions will be similar in spirit to the present version, but may differ in detail to address new problems or concerns.

   Each version is given a distinguishing version number. If the Program specifies a version number of this License which applies to it and "any later version", you have the option of following the terms and conditions either of that version or of any later version published by the Free Software Foundation. If the Program does not specify a version number of this License, you may choose any version ever published by the Free Software Foundation.

10. If you wish to incorporate parts of the Program into other free programs whose distribution conditions are different, write to the author to ask for permission. For software which is copyrighted by the Free Software Foundation, write to the Free Software Foundation; we sometimes make exceptions for this. Our decision will be guided by the two goals of preserving the free status of all derivatives of our free software and of promoting the sharing and reuse of software generally.

No Warranty

11. Because the program is licensed free of charge, there is no warranty for the program, to the extent permitted by applicable law. Except when otherwise stated in writing the copyright holders and/or other parties provide the program "as is" without warranty of any kind, either expressed or implied, including, but not limited to, the implied warranties of merchantability and fitness for a particular purpose. The entire risk as to the quality and performance of the program is with you. Should the program prove defective, you assume the cost of all necessary servicing, repair or correction.

12. In no event unless required by applicable law or agreed to in writing will any copyright holder, or any other party who may modify and/or redistribute the program as permitted above, be liable to you for damages, including any general, special, incidental or consequential damages arising out of the use or inability to use the program (including but not limited to loss of data or data being rendered inaccurate or losses sustained by you or third parties or a failure of the

program to operate with any other programs), even if such holder or other party has been advised of the possibility of such damages.

End of Terms and Conditions

References

[1] Thompson J, Gopal S: **Genetic algorithm learning as a robust approach to RNA editing site prediction**. *BMC Bioinformatics* 2006, **7**:145.

[2] Thompson J, Gopal S: **Correction: Genetic algorithm learning as a robust approach to RNA editing site prediction**. *BMC Bioinformatics* 2006, **7**:145.
